# Transforming growth factor-β1 up-regulates connexin43 expression in osteocytes via canonical Smad-dependent signaling pathway

**DOI:** 10.1042/BSR20181678

**Published:** 2018-12-14

**Authors:** Wenjing Liu, Yujia Cui, Jianxun Sun, Linyi Cai, Jing Xie, Xuedong Zhou

**Affiliations:** State Key Laboratory of Oral Diseases, West China Hospital of Stomatology, Sichuan University, Chengdu, China

**Keywords:** Connexin 43, Gap junction, Osteocyte, Smad3, Smad4, TGF-β1

## Abstract

Connexin 43 (Cx43)-mediated gap junctional intercellular communication (GJIC) has been shown to be important in regulating multiple functions of bone cells. Transforming growth factor-β1 (TGF-β1) exhibited controversial effects on the expression of Cx43 in different cell types. To date, the effect of TGF-β1 on the Cx43 expression of osteocytes is still unknown. In the present study, we detected the expression of TGF-β1 in osteocytes and bone tissue, and then used recombinant mouse TGF-β1 to elucidate its effect on gap junctions (GJs) of osteocytes. Our data indicated that TGF-β1 up-regulated both mRNA and protein expression of Cx43 in osteocytes. Together with down-regulation of Cx43 expression after being treated with TGF-β type I receptor inhibitor Repsox, we deduced that TGF-β1 can positively regulate Cx43 expression in osteocytes. Thus we next focussed on the downstream signals of TGF-β and found that TGF-β1-mediated smads, Smad3 and Smad4, to translocate into nucleus. These translocated signal proteins bind to the promoter of *Gja1* which was responsible for the changed expression of Cx43. The present study provides evidence that TGF-β1 can enhance GJIC between osteocytes through up-regulating Cx43 expression and the underlying mechanism involved in the activation of Smad-dependent pathway.

## Introduction

Osteocytes, which make up >95% of bone cells, had been considered a relatively inactive cell for many years as there has been a lack of clear understanding about their functions in the skeleton. Recent studies thought that osteocytes are the master orchestrators of differentiation and function of osteoblasts and osteoclasts. It has been revealed that although osteocytes reside in the mineralized bone matrix, they connect to cells on the bone surface and to the vasculature through stretching their dendritic processes [1]. This cell-to-cell and cell-to-matrix communication is mediated by gap junctions (GJs) and hemi-channels. GJs at the tips of osteocyte processes respond to extracellular stimuli and transmit the signal to other bone cells and regulate the exchange of small molecules amongst osteoblast lineage cells [2,3].

GJs consist of two pairing hemi-channels (connexons, which are composed by six connexin protein hexamers in the plasma membrane) [4]. These channels are passages to transfer molecules smaller than 1.2 kDa including small ions, metabolites, ATP, prostaglandin, and inositol 1, 4, 5-trisphosphate (IP_3_). Human beings have at least 21 isoforms of connexin [5] and *Mus musculus* has 20 orthologous connexins [6]. Amongst those Cx transmembrane proteins, connexin43 (Cx43) is the most highly expressed in bone [7,8]. Cx43-mediated gap junctional intercellular communication (GJIC) serves important roles in the skeletal network, including participation in mechanotransduction [9], endocortical bone resorption and bone remodeling, regulating osteocyte survival, and so on [10]. This vital protein can be affected by some cytokines, such as interleukin, tumor necrosis factor-α (TNF-α), and transforming growth factor-β [11].

Transforming growth factor-β superfamily (TGF-β superfamily) is involved in a majority of cellular processes and plays fundamental role in regulating survival [12]. This superfamily consists of two general branches: (i) bone morphogenic protein (BMP)/growth differentiation factor (GDF) and (ii) the TGF-β/activin/nodal branch/mullerian-inhibiting substance (MIS) or anti-mullerian hormone [13,14]. Transforming growth factors-β (TGF-βs) are secreted polypeptides and mainly stored as a latent complex in the extracellular matrix and exists in at least three isoforms: TGF-β1, TGF-β2, and TGF-β3. Amongst them, TGF-β1 is the most abundant growth factor in human bone. It is known that TGF-β1 exhibits diverse function in regulating cells of skeletal muscle system both during embryogenesis and in adult organism [15]. It has been proven to be an inducer of osteoblast proliferation [16], bone marrow-derived adult human mesenchymal stem cells differentiation [17], and overexpression of TGF-β1 enhances chondrogenic differentiation and proliferation of human synovium-derived stem cells [18]. TGF-β1 also could mediate expressions of LOXs in ACL and MCL fibroblasts [19].

However, to the best of our knowledge, the possible effect of TGF-β1 on osteocytes especially on their GJIC and expression of Cx43 have not been reported so far. Recently, there are studies indicating that TGF-β1 up-regulates the expression of Cx43 in human granulosa cells [20] and trophoblast cells [21]. In contrast, some research also reported that TGF-β1 down-regulates Cx43 expression in cultured smooth muscle cells from human detrusor [22] and in rat hepatic stellate cells [23]. These conflicting roles of TGF-β1 in the regulation of Cx43 in different types of cells intrigue us to study how is the effect of TGF-β1 on Cx43 expression and GJIC in osteocytes. Thus we explored the changes of Cx43 in osteocyte after TGF-β1 administration and subsequently we investigated the possible mechanism to be involved in.

## Materials and methods

### Animals

The animal materials used for the present study were obtained according to ethical principles and all protocols were approved by Institutional Review Board (IRB) of Sichuan University (IRB at the West China Hospital of Stomatology, No.WCHSIRB-D-2017-029). Two-month-old male C57BL mice were obtained from the Experimental Animal Center of Sichuan University and housed in pathogen-free facilities under a 12-h light and 12-h dark cycle.

### Cell culture

Murine MLO-Y4 cell line (American Type Culture Collection, Mannasas, VA), late osteoblastic cell line, which has characteristics similar to osteocytes, was used in the present study. Maintenance medium was DMEM (high-glucose DMEM, 0.1 mM non-essential amino acids, 4 mM l-glutamine) supplemented with 10% FBS, 1% penicillin–streptomycin solution. Cells were cultured at 37°C in a 5% CO_2_ incubator till confluency.

### Preparation of tissue sample

Cortical bone was harvested from the hind leg of 2-month-old male C57BL mice (both tibia and femur with no joint head). First, the mouse was killed and sterilized. Then the hind leg was taken out and immediately moved on to ice. Intact femur and tibia were extracted with the attached tissues to be completely removed (remaining cortical bone part only). Soft tissues in bone marrow cavity were then rinsed with PBS thoroughly by using an injector. Then the cortical bone parts were crushed by using liquid nitrogen. The fine residue was collected for PCR assays.

### Quantitative real-time PCR

Total RNAs were extracted from osteocyte and bone by using the RNeasy Plus Mini Kit (Qiagen, CA, U.S.A.) according to the manufacturer’s protocols. Dissolved in RNase-free water, RNA samples were quantitated by the NanoDrop® spectrophotometer (Nano Spectrophotometer 2000c, Thermo Fisher Scientific, U.S.A.). To obtain cDNA, RNA was reverse-transcribed by the cDNA synthesis kit (K1621-RevertAid, Mbi, MD, U.S.A.). Quantitative real-time PCRs were performed with the SYBR Premix ExTaq II PCR Kit (TAKARA, Shiga, Japan) using an iCycler (Bio-Rad) according to the manufacturer’s protocol. The PCRs contained 1.0 μM for each primer pairs (Supplementary Table S1 for TGF-β superfamily and Cx43, Supplementary Table S2 for the primer pairs of TGF-β superfamily receptors. BLAST was used to search for all primer sequences to ensure gene specificity) and 1 μl cDNA sample in a 25-μl volume. The PCR program is composed of a 5-s pre-incubation at 95°C. Amplification was achieved with 39 cycles of 5 s denaturation at 95°C, 30 s annealing at 60°C, and 5 s extension at 72°C. All experiments were performed in triplicate. Relative expression was calculated using a ΔΔ*C*_t_ method by normalizing with *gapdh* as the internal control.

### Semi-quantitative RT-PCR

Semi-quantitative RT-PCR was used to evaluate the mRNA expression levels of TGF-β superfamily in the way of agarose gel images. Semi-quantitative PCRs were performed with PCR Kit (Mbi, MD, U.S.A.) using a thermocycler (Bio-Rad, CA, U.S.A.). The reactions were performed in a 25-μl volume containing 1 μl cDNA sample, 1 μl forward primer, and 1 μl reverse primer. Products were resolved by 2% agarose gel electrophoresis in Tris-borate/EDTA buffer and visualized by staining with Ethidium Bromide.

### Protein extraction and Western blotting analysis

The expression level of Cx43 and essential proteins (Smad3, Smad4, p-Smad3) involved in the Smad signaling pathway were analyzed by Western blot. Briefly, MLO-Y4 cells were cultured for 24 h and then respectively treated with recombinant mouse TGF-β1 (0.1, 1, 5, 10 ng/ml, p04202, R&D Systems, U.S.A.) for 24 and 36 h, with Repsox (ab142139, Abcam, Cambridge, U.K.) (25, 50 μM) and 0.01% (v/v) DMSO for 24 h. Control groups were set up with no treatment. Cells were washed three times with ice-cold PBS, and lysed in lysis buffer containing protease inhibitor (1% (v/v) PMSF, Sigma). The concentrations of samples were determined by BCA assay (Beyotime, Shanghai, China). Proteins were separated by SDS/PAGE and transferred to PVDF membranes (PALL, U.S.A.). Membranes were blotted with 5% milk for 1 h and then incubated overnight at 4°C with the corresponding primary antibodies (β-actin, 1:2000, sc-47778; Cx43, 1:3000, #11370; Smad3,1:3000, #28379; Smad4, 1:5000, #40759; p-Smad3, 1:2000, #52903; Abcam, Cambridge, U.K.), then added corresponding secondary antibody (m-IgG_К_BP-HRP, 1:4000, sc-516102; mouse anti-rabbit IgG-HRP, 1:2000, sc-2357) and incubated for 2 h. β-actin was used as the internal control. The immunocomplexes were visualized with Super Signal reagents (Pierce, Rockford, IL). The ImageJ software (NIH, Bethesda, MD, U.S.A.) was used for densitometric analyses of the blots. All experiments were repeated three times and the most representative images were selected to present in the ‘Results’ section.

### Cell counting kit-8 assay

MLO-Y4 cells were seeded on the 96-well plates at a density of 2000 cells per well. The cells were incubated at 37°C in a 5% CO_2_ incubator for 12 h after cell adherence and then treated with 10 μl different concentrations of TGF-β1 and continued to incubate for 24 h. Each well was added with 20 μl of cell counting kit-8 (CCK-8) reaction solution and then incubated at 37°C for 2 h in the dark. Absorbance of each well was examined by reading the optical density value at 450 nm.

## Wound-healing assay

We applied a linear wound 10 × 1.4 mm (length × width) by scraping the osteocytes with a pipette tip followed by washing with PBS to remove cell debris as previously reported [24], then changed fresh culture medium contained with TGF-β1 (0.1, 1, 5, 10 ng/ml) to investigate its effect on osteocyte wound repair *in vitro*. Images were taken at 0 h (control) and 24 h after scraping, respectively. The mobility ratio is calculated by migrated cell area/scraped area. The area was quantitated by ImageJ software (NIH, Bethesda, MD, U.S.A.). Also we set up control added fresh medium without TGF-β1 after scraping.

## Immunofluorescence and confocal laser scanning microscopy

The effect of TGF-β1 and Repsox on the expression of Cx43, Smad3, and Smad4 was detected by confocal laser scanning microscopy (CLSM). Osteocytes were cultured in Petri dishes specified for confocal laser microscopy for 12 h. Then 5 ng/ml TGF-β1 and 50 μM Repsox were added into the culture medium respectively as experimental groups, continued to incubate for 24 h. To detect the effect of TGF-β type I receptor inhibitor Repsox on the TGF-β1 modulation of Cx43, osteocytes were pretreated with 50 μM Repsox for 6 h and then treated with 5 ng/ml TGF-β1 for 24 h. The culture medium was discarded and PBS was used to wash the samples thrice. Then cell samples were fixed with 4% cold paraformaldehyde solution, permeabilized with 0.5% Triton X-100 (Beyotime, Shanghai, China) for 10 min, and blocked with 5% BSA for 1 h. Anti-Cx43 (1:200; Abcam, Cambridge, U.K.), Smad3 (1:200; Abcam, Cambridge, U.K.), and Smad4 (1:200; Abcam, Cambridge, U.K.). Rabbit monoclonal antibodies were used to incubate the samples overnight at 4°C, and a fluorescence–conjugated secondary labeled anti-rabbit antibody (10 μg/ml, Alexa Fluor ®647, Life Technology, Grand Island, NY, U.S.A.). Nuclei were counterstained with DAPI (D9542, Sigma, U.S.A.) and phalloidine (6 μM, Invitrogen, CA) was applied to stain the cytoskeleton. Confocal images were captured using a confocal microscopy system (Olympus, FV3000, Japan). All experiments were repeated at least three times.

## Scrape loading and dye transfer assay

The scrape loading/dye transfer (SL/DT) technique, which relies on the introduction of small molecular (MW < 900) dyes (Lucifer Yellow, MW457, L0259, Sigma) and tracing their intercellular movement through GJs, is used to assess the effect of TGF-β1 on the GJIC between osteocytes. Lucifer cannot get into intact cells whereas it can be introduced into cells through transient tear in the cell membrane produced by scrape loading. The adherent cells were treated with TGF-β1 for 6 h, then were rinsed with CaMg-PBS and scraped by a surgical blade prior to the addition of fluorescent dye (1 mg/ml Lucifer Yellow). After incubation for 2 min at room temperature, LY dye was aspirated and cells were rinsed to remove all extracellular fluorescence. We monitored the travel of the LY dye through several adjacent cell layers for 7 min.

## Bioinformatics analysis

We got the gene information such as GenBank ID and the promoter sequence (∼2000 bp) before transcriptional starting sites (TSS) of Gja1 from NCBI resources (https://www.ncbi.nlm.nih.gov/) and BioGPS (http://biogps.org/#goto=welcome). Moreover, the binding site sequences predicted at the promoters of Gja1 were obtained through the tool PROMO (http://alggen.lsi.upc.es/cgi-bin/promo_v3/promo/promoinit.cgi?dirDB=TF_8.3).

## Statistical analysis

The results are presented as the mean ± S.E.M. of at least three individual experiments and plotted with (GraphPad Prism Inc., San Diego, CA, U.S.A.). Data were analyzed by one-way ANOVA followed by Tukey’s protected least-significant difference post-hoc test for multiple comparisons. The critical significance level was set to be *P*<0.05.

## Results

### Gene expression of TGF-β superfamily in osteocytes and bone tissue

**Figure 1:**
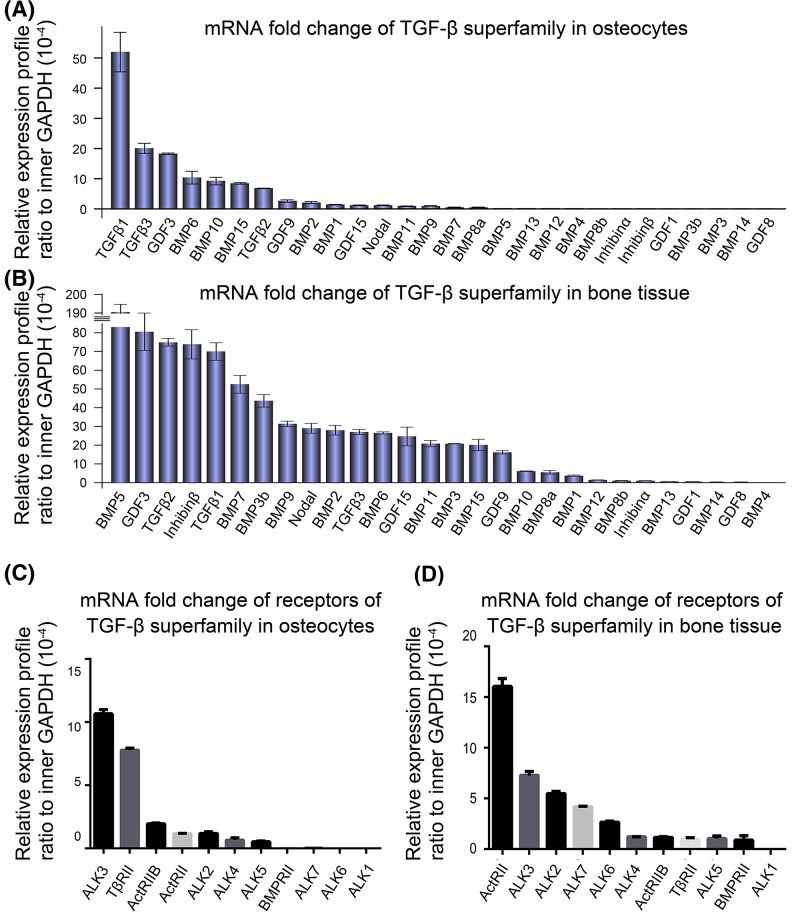
Gene profile of TGF-β superfamily and receptors of TGF-β superfamily in mouse osteocyte and bone tissue (**A**,**B**) Quantitative real-time PCR screen showing higher mRNA expression of TGF-β1 amongst TGF-β superfamily in both osteocyte cell line (A, upper) and bone tissue (B, lower). The results were based on the three independent experiments (*n*=3). (**C**,**D**) Quantitative real-time PCR screen showing mRNA levels of receptors of TGF-β superfamily in both osteocyte cell line (C) and bone tissue (D). The results were based on the three independent experiments (*n*=3).

We investigated the expression of TGF-β superfamily members and their receptors in both osteocytes and bone tissue by quantitative real-time PCR (Figure 1). The results showed that TGF-β1 exhibited the highest expression in osteocytes amongst TGF-β superfamily members. Moreover, gene expression of TGF-β2 and TGF-β3 are relatively high (Figure 1A). As for bone tissue, gene expression of TGF-β1 also ranks amongst the top five (Figure 1B). The mRNA levels of TGF-β superfamily receptors were also shown in both osteocyte cell line (Figure 1C) and bone tissue (Figure 1D). In addition, we further reconfirmed the expression of TGF-β superfamily genes by semi-quantitative reverse transcription PCR (Supplementary Figure S1). We detected four subfamilies, i.e. BMP subfamily, GDF subfamily, transforming growth factor (TGF) subfamily, and Inhibins and Nodal subfamily [25]. The expressions of BMP subfamily (Supplementary Figure S1A), GDF subfamily (Supplementary Figure S1B), TGF subfamily (Supplementary Figure S1C), and Inhibins and Nodal subfamily (Supplementary Figure S1D) by semi-quantitative reverse transcription PCR were consistent with the results of qPCR (Figure 1A). Agarose gel images showed that TGF-β1, TGF-β2, and TGF-β3 were also relatively high (Supplementary Figure S1C).

### TGF-β1 enhances GJ formations of osteocytes

**Figure 2:**
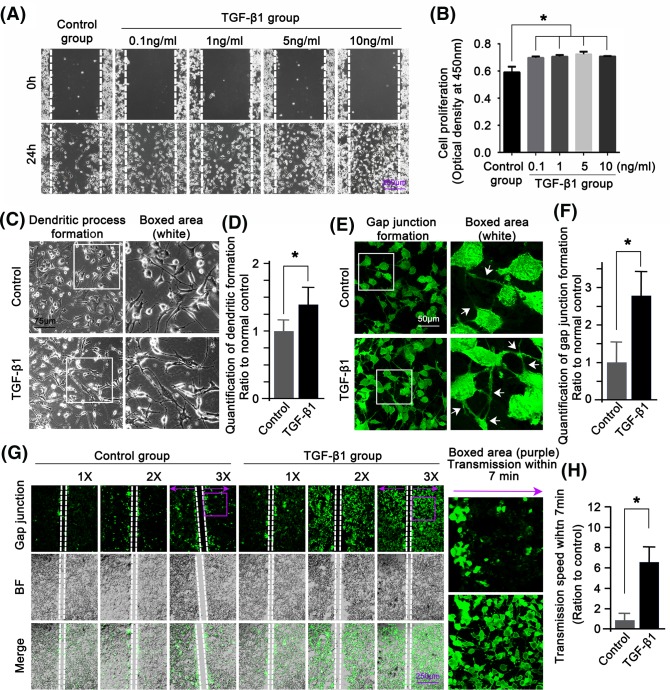
The TGF-β1 promotes cell–cell GJ of osteocytes (**A**) Representative images of scratch wound closure assay observed by phase-contrast microscopy at 20× magnification. The results were based on three independent experiments (*n*=3). (**B**) CCK-8 assay showing increased cell proliferation of osteocytes induced by increased dosages of TGF-β1. The results were based on the three independent experiments (*n*=3). *Significant difference was respected to the control group. ng denotes ng/ml. (**C**) Cell morphology assay showing the increase in dendritic processes in osteocytes induced by 5 ng/ml TGF-β1 by phase-contrast microscopy at 40× magnification. The results were based on three independent experiments (*n*=3). (**D**) Quantitation was done to confirm the increase in dendritic processes induced by TGF-β1. *Significant difference was respected to the control group (*P*<0.05). (**E**) The dye transfer (DT) assay showing the increased GJ formation in osteocytes induced by TGF-β1 (5 ng/ml) by CLSM at 40× magnification. The dye transfer images were collected at 10 min after Lucifer Yellow staining. The results were based on three independent experiments (*n*=3). (**F**) Quantitation was done to confirm the increase in GJ formation in osteocytes induced by TGF-β1. *Significant difference was respected to the control group (*P*<0.05). (**G**) The SL/DT assay further showing a cell density-dependent increase in GJs in osteocytes induced by TGF-β1 (5 ng/ml); 1×, 2×, and 3× represent multiplied cell densities. The images were collected at 7 min after Lucifer Yellow staining along the scrape. The boxed area further showed the different transmission speeds between the control and TGF-β1 group. The purple arrows showed transmission direction after Lucifer Yellow loading. (**H**) Statistical analysis showing the changes inf transmission speeds after Lucifer Yellow loading between the control and TGF-β1 group (5 ng/ml). Data are presented as mean ± S.E.M. (*n*=3). *P<0.05.

We first applied the scratch wound closure assay to show the migration rates of osteocytes after TGF-β1 treatment with different concentrations (Figure 2A). Osteocyte migration was shown to be significantly increased by TGF-β1 with different concentrations. CCK-8 assay then confirmed that the cell proliferation was also enhanced by TGF-β1 with different concentrations (Figure 2B). At a higher magnification observation with phase-contrast microscopy, we found that the dendritic processes of osteocytes were significantly increased and lengthened (Figure 2C). The increase in dendritic processes was up to 35% in the TGF-β1 treated group (5 ng/ml) compared with the normal control group (Figure 2D). It enables us to further deduce that the GJ changes amongst cells after TGF-β1 treatment. With the SL/DT assay, we first found that the GJ formations (white arrows) between osteocytes were significantly increased in the TGF-β1 (5 ng/ml) group relative to that in the normal control group (Figure 2E). The GJ formations induced by TGF-β1 were increased to be approximately 2.75-fold compared with the normal control group (Figure 2F). We then elucidated the GJ formations through Lucifer Yellow stain and found that GJ formations were shown to be correlated with cell density. Moreover, TGF-β1-induced GJ formations were much more in comparison with the non-treated control group at the same cell density (Figure 2G). Finally, the TGF-β1-induced GJ formations showed higher transmission speed of fluorescent dyes within 7 min (Figure 2H).

### TGF-β1 up-regulates GJs in osteocytes through the increase in Cx43

**Figure 3:**
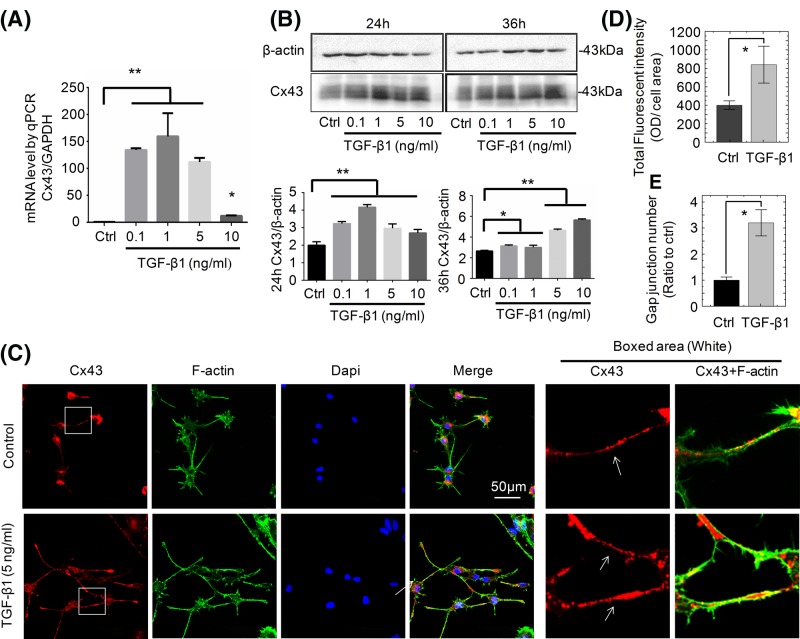
The TGF-β1 promotes GJ through the increase in Cx43 (**A**) mRNA expression of Cx43 by qPCR after being treated with different concentrations of TGF-β1. The results were based on three independent experiments (*n*=3). **P<0.01. (**B**) Western blot analysis of Cx43 upon exposure to TGF-β1 for 24 and 36 h. Quantitation was performed to confirm the protein changes (*n*=3). *P<0.05; **P<0.01. (**C**) Representative IF staining by CLSM showing the elevated expression of Cx43 and potential GJ in osteocytes in response to TGF-β1 (cytoskeleton, green; Cx43, red; nucleus, blue). The results were based on three independent experiments (*n*=3). White arrows showing that Cx43 distribution was along the dendritic processes. (**D**) The total fluorescent qualification was performed to show the expression changes of Cx43 induced by TGF-β1 (5 ng/ml). The results were based on three independent experiments (*n*=3). *P<0.05. (**E**) The qualification further indicated the changes of GJ numbers induced by TGF-β1 (5 ng/ml). *Significant difference was respected to the control group (*P*<0.05).

It has been reported that GJs consist of connexin protein hexamers in the plasma membrane and Cx43 is the most abundant member in connexin protein family [4]. To examine the effect of TGF-β1 on Cx43 in osteocytes, we cultured osteocytes with different concentrations of TGF-β1 (0.1, 1, 5, and 10 ng/ml) and found that TGF-β1 significantly up-regulated *Cx43* mRNA level at 24 h by qPCR (Figure 3A). Western blot assay next confirmed the protein increase in Cx43 (Figure 3B). Compared with the normal group, the Cx43 protein levels induced by TGF-β1 increased at 24 and 36 h. The blot quantitation verified that the up-regulation of Cx43 after TGF-β1 treatment was significantly increased compared with that in the control group (Figure 3B). To further explore the distribution of Cx43 in osteocytes induced by TGF-β1, we performed the immunostaining and found that Cx43 located mainly in the cytoplasm and dendritic processes. Especially, Cx43 was shown to be clustered as red fluorescent plaques along the dendritic processes between cells, which are the typical appearances of Cx43 channels in GJs (Figure 3C). After being treated with 5 ng/ml TGF-β1, the dendritic processes of osteocytes became longer and GJs plaques between cells were shown to be obviously more and brighter as indicated by the arrows in Figure 3C. The total fluorescent qualification indicated that the expression of Cx43 in the TGF-β1-treated group was significantly increased relative to that in the normal control group (approximately up to 2.1-folds, Figure 3D). The qualification on GJ numbers further showed that TGF-β1 could significantly enhance the GJ formation (Figure 3E).

### Inhibition of endogenous TGF-β1 decreases the expression of Cx43 in osteocytes

**Figure 4:**
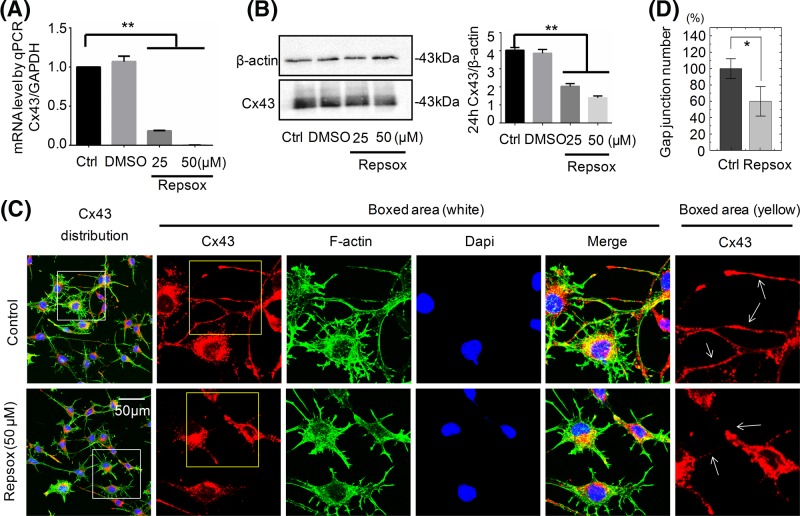
Repsox, an inhibitor of TGF-β type I receptor, reduces the expression of Cx43 (**A**) qPCR showing down-regulated mRNA expression of Cx43 after being treated with Repsox. The results were based on the three independent experiments (*n*=3). **P<0.01. (**B**) Western blot assay showing the expressions of Cx43 upon exposure to Repsox for 24 h. Quantitation was performed to confirm the protein changes (*n*=3). **P<0.01. (**C**) Representative IF staining by CLSM showing the decreased Cx43 in osteocytes in response to Repsox (cytoskeleton, green; Cx43, red; nucleus, blue). The results were based on three independent experiments (*n*=3). (**D**) Quantitation of GJ number by ImageJ was performed to confirm the changes after Repsox treatment (*n*=3). *P<0.05.

Repsox, a specific chemical reprogramming tool and ATP-competitive inhibitor of TGF-β receptor 1 kinase (ALK5) [26,27], can block TGF-β receptor signaling. We here used it to explore its influence on Cx43 in osteocytes. As shown in Figure 4A, treatment with 25 and 50 μM Repsox down-regulated *Cx43* mRNA levels in osteocytes (DMSO, which was used as the vehicle control, had almost little effect on the expression of Cx43). Similarly, at protein level, the expression of Cx43 in osteocytes was decreased after treatment with Repsox for 24 h and this down-regulation effect was statistically significant in osteocytes treated with 25 and 50 μM Repsox (Figure 4B). We further investigated the effect of Repsox on Cx43 by immunostaining assay. The results showed that not only did the dendritic processes become shorter, but also the Cx43 expression and GJs between cells were reduced as indicated by the arrows in Figure 4C. The reduction in GJ number could reach up to 40% (Figure 4D).

### TGF-β1 modulates Cx43 through nuclear translocation of Smads and resultant binding to the promoter of Cx43

**Figure 5:**
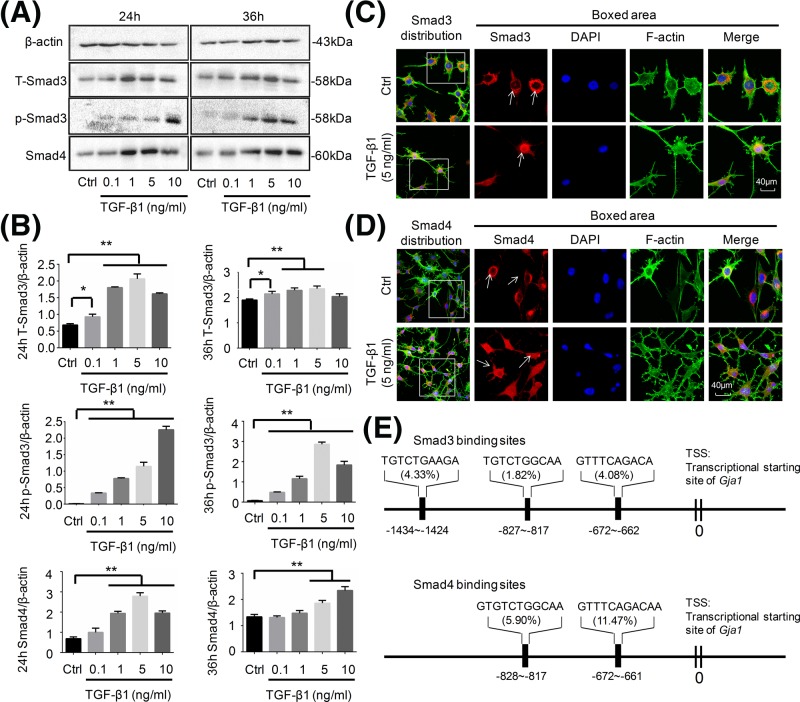
TGF-β1 mediates Cx43 via transducing signals, Smad3 and Smad4, by direct binding signals to the promoter of *Cx43* gene (**A**) Western blot assay showing the expression of Smad3, p-Smad3, and Smad4 upon exposure to TGF-β1 for 24 and 36 h. The results were based on three independent experiments (*n*=3). (**B**) Quantitations were performed to confirm the protein changes in (A) (*n*=3). *P<0.05, **P<0.01. (**C**,**D**) Representative IF staining by CLSM showing the nuclear translocation of Smad3 (upper) and Smad4 (lower) in osteocytes in response to TGF-β1 (cytoskeleton, green; Samd3 and Smad4, red; nucleus, blue). The results were based on three independent experiments (*n*=3). (**E**) Bioinformatics showing the binding sites of nuclear translocated-Smad3 and -Smad4 to the promoter of Cx43 gene (*Gja1*, GenBank name). Smad3 showed three potential binding sites in the promoter of *Gja1*, and the sites of sequences were located at 1434–1424, 827–817, and 672–622 kb before TSS of *Gja1*. Smad4 showed two binding sites in the promoter of Gja1, and the sites of sequences were located at 828–817 and 672–621 kb before TSS of *Gja1*.

The above results prompted us to explore the mechanism by which TGF-β1 up-regulated Cx43 expression. To date, Smad-dependent pathway is proved to be the main signaling pathway in response to TGF-β1-mediated osteoblast and chondrocyte differentiation [28]. To test whether the Smad-dependent pathway is of great importance in the TGF-β1-mediated Cx43 in osteocytes, the expression of one of the receptor-activated Smads (R-Smads), Smad3, and the common Smad (Co-Smad), Smad4, were explored (Figure 5A). Total Smad3 was significantly up-regulated after treatment with 0.1, 5, and 10 ng/ml TGF-β1 for 24 h, and also increased at 36 h. Interestingly, the expressions of p-Smad3 were all significantly at high levels in all treatment groups compared with the control group. Moreover, quantitation showed that Smad4 in osteocytes treated with 1, 5, and 10 ng/ml TGF-β1 at 24 h and in those treated with 5, 10 ng/ml TGF-β1 at 36 h exhibited increasing expression (Figure 5B). We next explored the distribution of Smad3/4 after TGF-β1 induction and found that the TGF-β1-induced Samd3/4 were mainly accumulated at/around nucleus (Figure 5C,D). By using the bioinformatics, we found that samd3/4 translocated into nucleus by TGF-β1 were shown to have the binding sites in the promoter of *Cx43* gene, namely GJ α 1, *Gja1.* Besides, Smad3 and Smad4 were shown to have two identical binding sites in the promoter of *Gja1* (Figure 5E), which corresponded to previous reports that Smad3 and Smad4 exerted their effect by forming complexes and then translocating into the nucleus [25].

**Figure 6:**
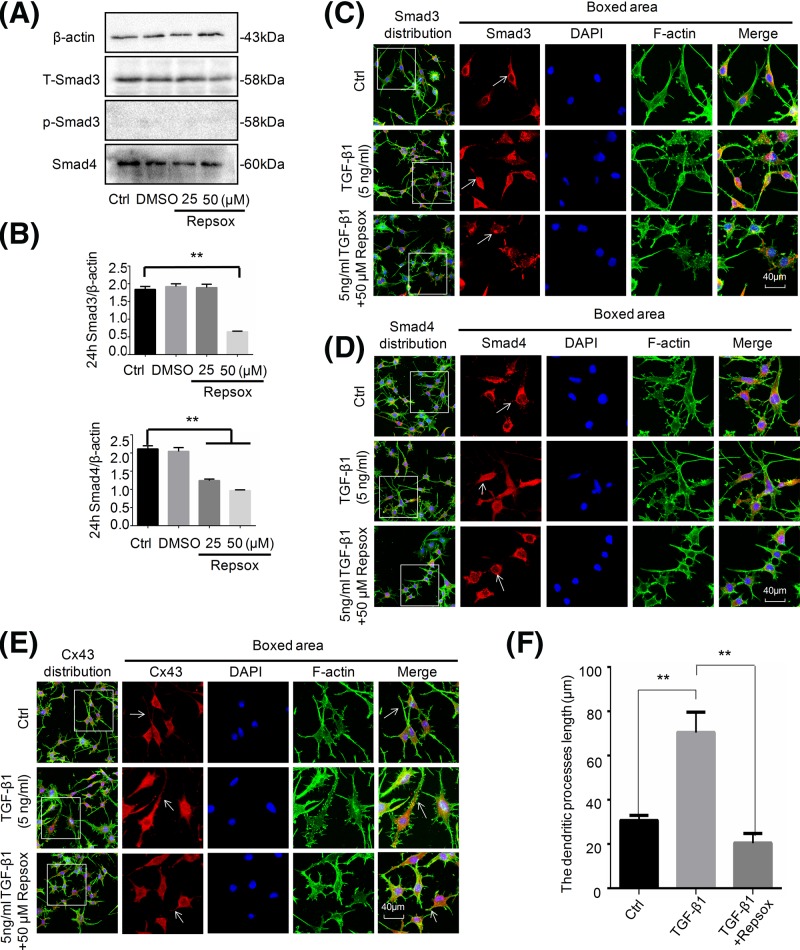
Repsox represses translocation of Smad3 and Smad4 into nucleus resulting in attenuation of the effect of TGF-β1 on Cx43 (**A**) Western blot assay showing the expression of Smad3, p-Smad3, and Smad4 upon exposure to Repsox for 24 h. The results were based on three independent experiments (*n*=3). (**B**) Quantitation was performed to confirm the protein changes in (A) (*n*=3). **P<0.01. (**C**,**D**) Representative IF staining by CLSM showing reduced translocation of Smad3 (C) and Smad4 (D) in osteocytes after pretreatment with 50 μM Repsox for 6 h and then treated with 5 ng/ml TGF-β1 for 24 h compared with the group only treated with TGF-β1 (cytoskeleton, green; Smad3 and Smad4, red; nucleus, blue). The results were based on the three independent experiments (*n*=3). (**E**) Representative IF staining by CLSM showing that Repsox attenuated the Cx43 expression even in TGF-β1-treated osteocytes. (**F**) Quantitation further showing the changes in dendritic processes of osteocytes pre-incubated with Repsox after treatments of TGF-β1 (*n*=3). **P<0.01.

We next detected the changes in Smad3 and Smad4 in osteocytes after Repsox treatment for 24 h and found that Repsox could significantly reduce Smad3 and Smad4 signals in osteocytes (Figure 6A,B). We further used CLSM and found that TGF-β1-induced nuclear translocations of Smad3 (Figure 6C) and Smad4 (Figure 6D) were all attenuated by Repsox (Repsox was used to pre-incubate osteocyte for 6 h before TGF-β1 treatment). We further explored the influence of classic signal pathway, Smads, on GJs by characterizing the Cx43 expression by CLSM. We found that Repsox reduced the expression of Cx43 in TGF-β1-treated osteocytes (Figure 6E). The quantitation on dendritic processes also showed that the effect of TGF-β1 on dendritic processes of osteocytes were reduced in Repsox pre-incubated osteocytes (Figure 6F).

## Discussion

Cells need to communicate with each other, and GJ is one of the quickest manners that directly connect the cytoplasm of adjacent cells. However, the exact mechanism which regulates GJs is still not well known. As far as we know, the present study, for the first time, found that TGF-β1 has the capacity to mediate up-regulation of *Cx43* mRNA and protein as well as the GJs of osteocytes. Moreover, the expression of endogenous TGF-β1 was relatively high in osteocytes and bone tissue. By applying recombinant mouse TGF-β1, we found that TGF-β1 promotes the proliferation and migration of osteocytes which are consistent with previous studies on the effect of TGF-β1 on proliferation of fibroblasts [29] and airway smooth muscle cells [30].

It has been reported that TGF-β enhanced cell proliferation of osteoblasts through activation of extracellular signal-regulated kinases (ERKs) [31]. In addition, elevated Cx43 in osteoblasts or osteocytes in turn increases ERK signaling and ERK signaling-related cell proliferation. Thus, we infer that this may explain the up-regulation of cell proliferation in the present study. In addition, we found that the transmission speed of LY dye increased after TGF-β1 treatment. We speculate that this is because TGF-β1 not only promoted cell proliferation, which resulted in an increase in cell density, but also up-regulated the expression of Cx43, thus increased GJs between cells, which allows only small molecules of dye to pass through.

The differentiation of pre-osteocytes and proliferation relies on signal transduction through GJs formed by connexins. Prior studies have noted the ubiquity of Cx43 in all bone cell types, deletion and dysfunction of Cx43 results in many diseases in mice [10]. It has been reported that absence of Cx43 in osteoblasts and osteocytes delayed the healing of fracture due to decreased bone formation and resorption [32]. A recent study found that ageing affects Cx43 expression and function as osteoblastic cells from old rats showed decreased GJ communication in response to PTH compared with cells from younger rats [33]. Our study indicates that TGF-β1 could promote *Cx43* mRNA and protein expressions; hence we deduce that this may be an approach to improve the viability and function of osteocyte in old bone.

**Figure 7:**
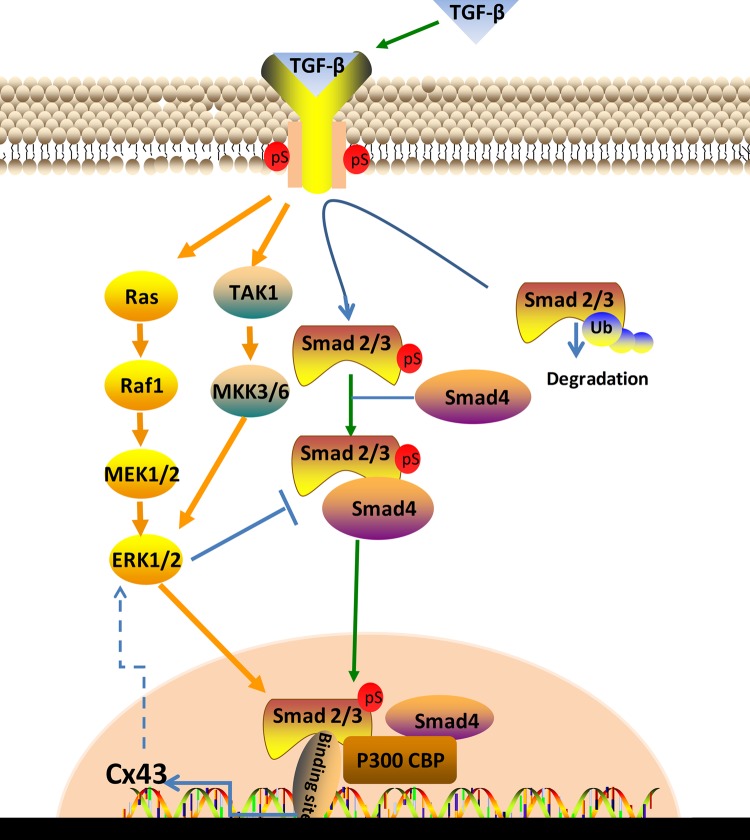
The schematic diagram elucidating the Smad-dependent pathway and non-Smad-dependent pathway involved in the changes of Cx43 expression induced by TGF-β1 The non-Smad-dependent pathway is predicted to be involved the changes of Cx43 but not in the present study.

TGF-β signaling consists of Smad-dependent pathway and non-Smad-dependent pathway. In these pathways, TGF-βs bind to TGF-β receptors and activate the downstream signaling cascade. In the current study, we found that upon employment of TGF-β1, expression of T-Smad3, p-Smad3, and Smad4 increased and nuclei translocations of the two proteins were enhanced. So we illuminate the mechanism from sensing active extracellular stimuli and delivering signals into osteocyte cytoplasm, to affect phenotype and function as follows. Trigged by the complex comprising TGF-β1 and TGF-β receptors, R-smad (Smad2 or Smad3) which exist in the cytoplasm, were phosphorylated and attract C-Smad (Smad4) to form complexes, those complexes subsequently translocated into the nuclei, where they recruit co-factors to regulate target gene Cx43 expression [28]. As for the TGF-βs signaling requires binding to type I and type II receptors, we used TGF-βR1/ALK5 inhibitor to explore the involvement of Smads in the effect of TGF-β1 on Cx43 of osteocytes. The results of Western blot showed that T-Smad3, and smad4 were down-regulated by 50 μM Repsox whereas p-smad3 exhibited no change. Furthermore, no nuclei translocation of Smad3 and smad4 were discovered by CLSM. The results confirmed our prefigured Smad-dependent signaling pathway involved in the TGF-β1 induced up-regulation of Cx43. The mechanism of TGF-β1 affecting the Cx43 expression through Smad-dependent signaling pathway was described in Figure 7. By bioinformatics analysis, we can verify that after activation by TGF-β1, the Smad3 and Smad4 complex translocate into nuclei where it has binding sites at the *Cx43* gene thus affecting the expression of Cx43.

Apart from the Smad-dependent pathway, TGF-β1 also initiates non-Smad-dependent pathways including phosphorylation of TAK1 and then activate the MKK-p38 MAPK or MKK-ERK1/2 signaling cascade as well as activating Ras-induced ERK pathway (Raf-MEK-ERK) [27,34]. Because the expression of Cx43 was decreased rather than abolished after treatment with Repsox, we assume that non-Smad-dependent pathway may play a part or role in the regulation of Cx43 induced by TGF-β1. Previous research revealed that the activation of ERK1/2 leads to increased TGF-β1-induced Cx43 expression [21]. In addition, Chis et al. found that ERK phosphorylated the linker region of nuclear localized Smads, enhanced Smad-mediated transcriptional activity [35], and increased duration of Smad target gene transcription [36]. Whereas some research pointed out that within the linker region of Smad2 and Smad3 are several potential ERK phosphorylation sites and those sites may inhibit Smad nuclear translocation and signaling [37]. Due to the two mutually exclusive functions of ERK, the mechanism of ERK signaling pathway is complicated and more evidence are needed to clarify the precise role of ERK1/2 in TGF-β1-induced Cx43 expression.

## Conclusion

We admit that some limitations exist in the present study. First, it is reported that there are more than 40 members in the TGF-β superfamily [37] whilr we profiled *TGF-β1* gene expression amongst the most common ones. Second, we only explored the role of Smad-dependent pathway in TGF-β1-induced up-regulation of Cx43, yet it is still necessary to make an insight into the accurate role of non-Smad-dependent pathway in TGF-β1-induced Cx43 expression.

In summary, our study demonstrated that TGF-β1 gene expression is relatively high in osteocytes and bone, and TGF-β1 could up-regulate the expression of Cx43 via Smad-dependent pathway in osteocyte. These results provide an approach to further understand cell–cell communication of osteocytes.

## Supporting information

**supplementary Figure 1:** 

**Table S1.** The primer pairs of TGF-β superfamily and Cx43 designed for qPCR detection.

**Table S2.** The primer pairs of TGF-β superfamily receptors for qPCR detection.

## Abbreviations

ACL: anterior cruciate ligament
BMP: bone morphogenetic protein
CCK-8: cell counting kit-8
CLSM: confocal laser scanning microscopy
Cx43: connexin 43
DMEM: Dulbecco’s Modified Eagle Medium
ERK: extracellular signal-regulated kinase
GAPDH: glyceraldehyde-3-phosphate dehydrogenase
GDF: growth differentiation factor
GJ: gap junction
*Gja1*: GJ α 1, GenBank name of Cx43
GJIC: gap junctional intercellular communication
IRB: Institutional Review Board
LOX: lysyl oxidase
LY: lucifer yellow
MAPK: mitogen-activated protein kinase
MCL: medial collateral ligament
SL/DT: scrape loading/dye transfer
TGF-β1: transforming growth factor-β1
