# Expression of Concern: Transforming growth factor-β1 up-regulates connexin43 expression in osteocytes via canonical Smad-dependent signaling pathway

**DOI:** 10.1042/BSR-20181678_EOC

**Published:** 2020-07-29

**Keywords:** Connexin 43, Gap junction, Osteocyte, Smad3, Smad4, TGF-β1

The Editorial Office has been made aware of potential issues surrounding the scientific validity of this paper, hence has issued an expression of concern to notify readers whilst the Editorial Office investigates.

