# Correction: Transforming growth factor-β1 up-regulates connexin43 expression in osteocytes via canonical Smad-dependent signaling pathway

**DOI:** 10.1042/BSR-20181678_COR

**Published:** 2020-10-16

**Keywords:** Connexin 43, Gap junction, Osteocyte, Smad3, Smad4, TGF-β1

This Correction follows an Expression of Concern relating to this article previously published by Portland Press.

The authors would like to make two corrections to their original article “Transforming growth factor-β1 up-regulates connexin43 expression in osteocytes via canonical Smad-dependent signaling pathway” (*Biosci Rep* (2018) 38 (6): BSR20181678, DOI: https://doi.org/10.1042/BSR20181678).

In Figure 2A, the authors would like to replace the cell image at 5 ng/ml (0h) with the corrected one presented here. In the original version (at 5 ng/ml, 0h) the image from the control group was used in error.

**Figure 2:**
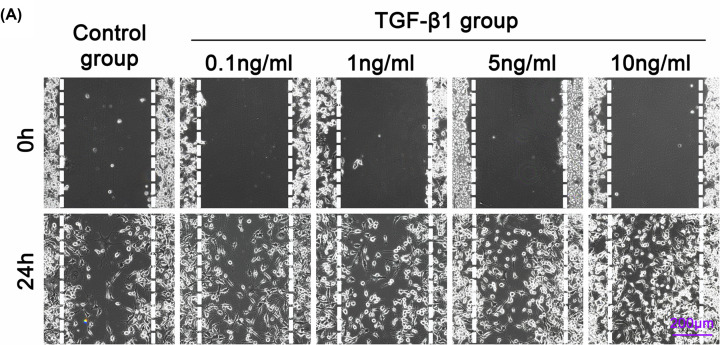
The TGF-β1 promotes cell–cell GJ of osteocytes (**A**) Representative images of scratch wound closure assay observed by phase-contrast microscopy at 20× magnification. The results were based on three independent experiments (*n* = 3).

In Figure 6D, the authors would like to replace the cell image of control group with the corrected one. In the original version of the Figure 6D, the image used in the Ctrl;Smad4 panel does not correspond to Smad4. The correct image from their reserved original data is used in the updated Figure 6 presented in this correction article.

**Figure 6:**
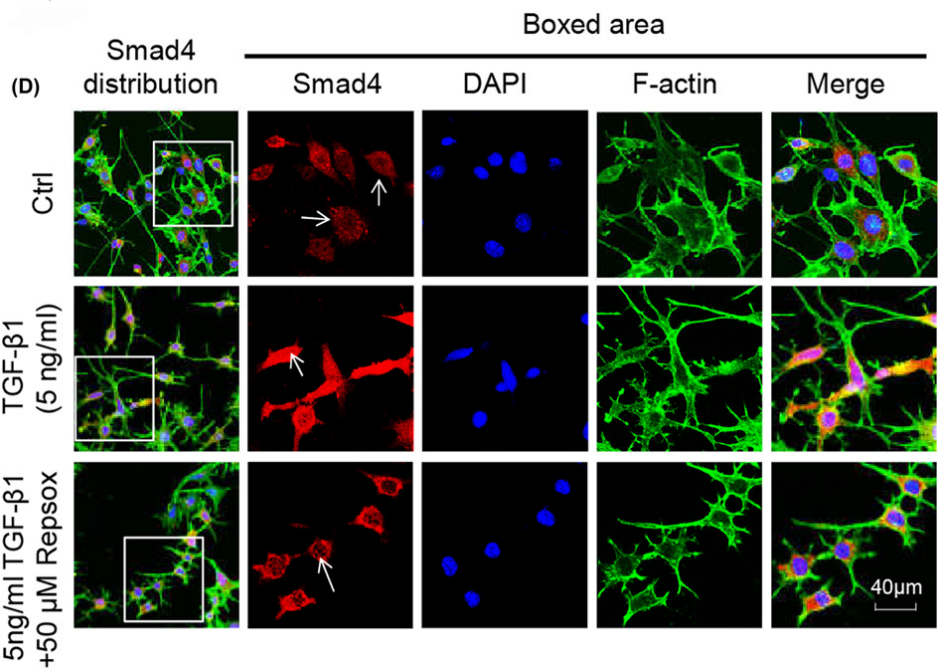
Repsox represses translocation of Smad3 and Smad4 into nucleus resulting in attenuation of the effect of TGF-β1 on Cx43 (**D**) Representative IF staining by CLSM showing reduced translocation of Smad4 in osteocytes after pretreatment with 50 μM Repsox for 6 h and then treatment with 5 ng/ml TGF-β1 for 24 h compared with the group only treated with TGF-β1 (cytoskeleton, green; Smad4, red; nucleus, blue). The results were based on the three independent experiments (*n* = 3).

The authors claim that these corrections do not affect the conclusions of this article. The authors also apologise for any inconvenience that these errors have caused to the readers of the paper.

