# Supplementary material for: Transforming growth factor-β1 up-regulates connexin43 expression in osteocytes via canonical Smad-dependent signaling pathway
Source: Biosci Rep. 2018 Dec 14;38(6):BSR20181678. doi: 10.1042/BSR20181678 (PMC6294634; doi:10.1042/BSR20181678)
Supplement: Supplementary file 1 [file bsr20181678_Supp1.pdf]

Supplementary Figure S1

Agarose gels using reversed transcript PCR products reconfirming the gene profile of TGF-β superfamily in osteocyte cell line. The representative mRNA expressions of BMP subfamily (A), GDF subfamily (B), TGF-βsubfamily (C) and Inhibins& Nodal subfamily (D) were shown. The representative images were based on the three independent experiments (n = 3). +, the standard reversed transcript PCR; mock, the reversed transcript-PCR without Taq DNA Polymerase shown as negative control.

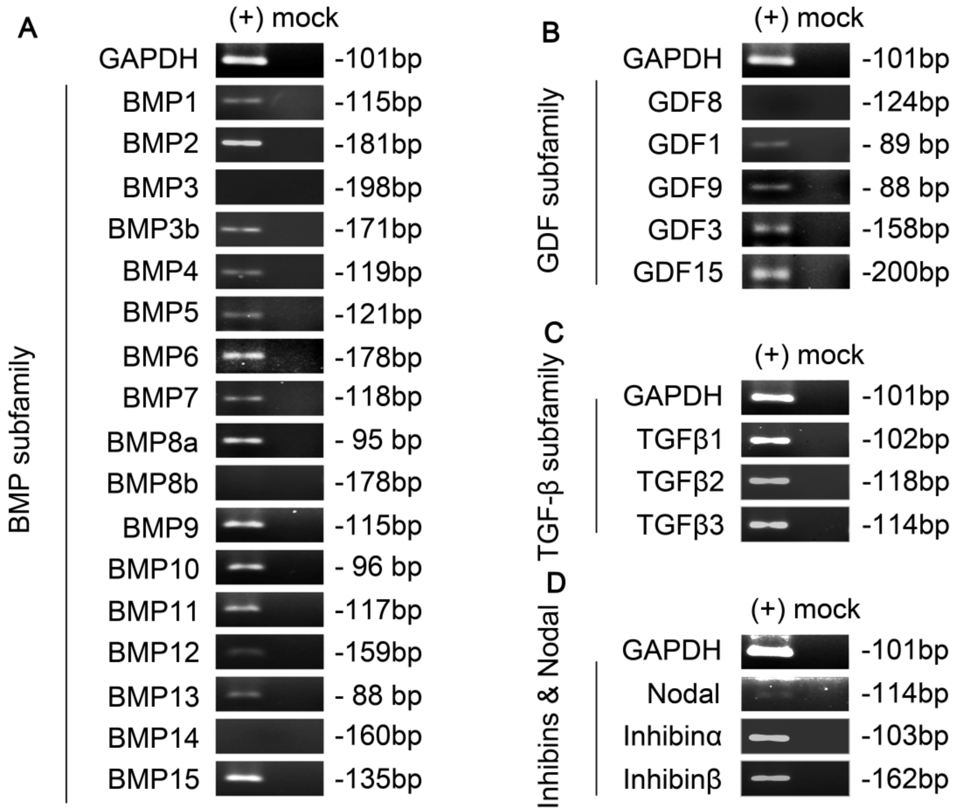

Supplementary Tables

Table S1.

Table S1. The primer pairs of TGF-β superfamily and Cx43 designed for qPCR detection.

mRNA	Accession number	Primer pairs (5'-3')		Product
GAPDH	NM_001289726.1	Forward	GGGTCCCAGCTTAGGTTCATC	87bp
		Reverse	AATCCGTTCACACCGACCTT	
BMP1	NM_009755.3	Forward	AGGGCGGCGAGAAAAGAAA	115bp
		Reverse	CTTCCCGTCCCGTTTCCTG	
BMP2	NM_007553.3	Forward	CCGCTGTCTTCTAGTGTTGCT	181bp
		Reverse	TCTCTGCTTCAGGCCAAACAT	
BMP3	NM_001310677.2	Forward	GGTCGAACCTCGGAACTGTG	198bp
		Reverse	TCCTCTACCCCGTGCAAAAA	
BMP3b	NM_145741.3	Forward	TCCCCATGCCCAAGATTGTC	171b
		Reverse	GGTACACCTTCAGAACCGCA	
BMP4	NM_001316360.1	Forward	CAGGAACCAATGAGACACCAT	119bp
		Reverse	TTTTCTTCCCGGTCTCAGGT	
BMP5	NM_007555.4	Forward	TTTCTGAGGAGTGGGGCTCT	121bp
		Reverse	GCTTGAAAGCTACAAGCGGG	
BMP6	NM_007556.3	Forward	TAATGTTCGCCTCCCCCAAC	178bp
		Reverse	TCCCCTCCATTCGGATGTCT	
BMP7	NM_007557.3	Forward	CCTATGGCCATGTCGCATCT	118bp
		Reverse	GCAGCCCAAGCTACTGAAGA	
BMP8a	NM_001256019.1	Forward	TGTGAGGGGGAGTGTGCTTT	95bp
		Reverse	CAGCAGGCTACTGTGGTACTGA	
BMP8b	NM_007559.5	Forward	GTCCGGGACTCCTATGGCTA	178bp
		Reverse	ACCGGTGCTCGGGATCG	
BMP9	NM_019506.4	Forward	TGCGCATGGTATGCCTAAGT	115bp
		Reverse	CCGCATACCCCAGTCATTGT	
BMP10	NM_009756.3	Forward	AACGCCAAGGGGAACTACTG	96bp
		Reverse	TTCATACCCAGGAGGAGCGA	

BMP11	NM_010272.2	Forward	GCACCCCTACCAAGATGTCC	117bp
		Reverse	CCACAACTTAGGAGCAGCCA	
BMP12	NM_001312876.1	Forward	GGTTCTGGCTTCAGGAACGG	159bp
		Reverse	CGCCGTTTCGTCTTGAGTTG	
BMP13	NM_013526.1	Forward	CCCCAACTGGTTTGCTCTCT	88bp
		Reverse	GCATTCCGACCGTTTCCTTG	
BMP14	NM_008109.3	Forward	GATCTGGCTGGGAGGTGTTC	160bp
		Reverse	AAGGCTTTCTCGTGGACCTG	
BMP15	NM_009757.5	Forward	CCGGACCAAGCACTTACCTT	135bp
		Reverse	CGAAGAACACTCCGTCCCTT	
TGF-β1	NM_011577.2	Forward	CACTCCCGTGGCTTCTAGTG	102bp
		Reverse	CTTCGATGCGCTTCCGTTTC	
TGF-β2	NM_001329107.1	Forward	AAAATCGACATGCCGTCCCA	118bp
		Reverse	CAAGGTACCCACAGAGCACC	
TGF-β3	NM_009368.3	Forward	CCTCAGGCTTTGGGATCTGG	114bp
		Reverse	TCATGTGTGAGCCCAGGAAC	
GDF1	NM_001163282.2	Forward	CAGCGGAGAATTGGATAGCA	89bp
		Reverse	GCAACATCTGCGCATAACTC	
GDF3	NM_008108.5	Forward	TGGTAGTCGATGAGTGTGGG	158bp
		Reverse	TGTGTGTAATTGTGGGGCTCAT	
GDF8	NM_010834.3	Forward	AGTACGACGTCCAGAGGGAT	124bp
		Reverse	TTGCCATCCGCTTGCATTAG	
GDF9	NM_008110.2	Forward	TGAAGTCAGTCTTCCACACCT	88bp
		Reverse	CATCTCCTCGTGCCAGTCTT	
GDF15	NM_001330687.1	Forward	CCTCCATCTTCTATCTGAGCCTG	200bp
		Reverse	CCATGTCGCTTGTGTCCTTTC	
Inhibinα	NM_001329843.1	Forward	GCCAGTTCCTAAGCCCCTCT	103bp
		Reverse	CACTGGATCAGTCCCGCTTG	
Inhibinβ	NM_008381.4	Forward	CACTTGCGGTCCTGAGTGAA	162bp
		Reverse	CAGTTTCGCCTAGTGTGGGT	
Nodal	NM_013611.5	Forward	AGGGGGAGTGCTGAAATTGG	114bp

		Reverse	TTAGCTCCAGCAGGCAGAAC	
Cx43	NM_010288.3	Forward	TGCACCTGGGGTGTTCATTT	126bp
		Reverse	GCCGCCTAGCTATCCCAAAA	

Table S2

Table S2. The primer pairs of TGF-β superfamily receptors for qPCR detection.

mRNA	Accession number	Primer pairs (5'-3')		Product
GAPDH	NM_001289726.1	Forward	GGGTCCCAGCTTAGGTTCATC	87bp
		Reverse	AATCCGTTCACACCGACCTT	
ALK1	NM_001277255.1	Forward	CGCGCAGATCGAGACCTTT	167bp
		Reverse	GATTTCCTGCCATTCAGGGT	
ALK2	NM_001110204.1	Forward	AACATCCCACCAGAAACCCT	142bp
		Reverse	TAAGTCAAGCCGGGGTCTTTC	
ALK3	NM_009758.4	Forward	ACCAGACGGTGTTAATGCGT	160bp
		Reverse	AGGGCTCTGGTGTCTAGTGT	
ALK4	NM_007395.4	Forward	CGTGTCTACCATAACCGCCA	150bp
		Reverse	CACTGTGCGCTGGACAAAAA	
ALK5	NM_001312868.1	Forward	CGAGACAGGCCATTTGTATGTG	117bp
		Reverse	TGACTGCTTTTCTGTAGTTGGG	
ALK6	NM_001277216.2	Forward	GATGCTCTGAGGACGGCTCT	76bp
		Reverse	AGCACTTAGCGCAGGGATTA	
ALK7	NM_001033369.3	Forward	CCACAGGTCTGCCTCTCTTG	102bp
		Reverse	ATCTTCCGTGCCACACTTCC	
TβRII	NM_009371.3	Forward	CCGCTGCATATCGTCCTGTG	97bp
		Reverse	TGGATGCATCTTTCTGGGCTT	
ActRII	NM_007396.4	Forward	TGGTCCCATGAACTTGCACT	135bp
		Reverse	GGGTCAGAAGCGATGTTTTCA	
ActRIIB	NM_001313757.1	Forward	AGAAGTCACGTACGAGCCAC	165bp
		Reverse	CCCGGGTCCTCATGGATGTC	
BMPRII	NM_007561.4	Forward	TTTGCAATAGAGTCAGCACACA	183bp

Reverse	GAACACGTTTCTAAGGCAGTGG
